# Evaluation of the Bioactivity of Phenolic Compounds from the *Sargassum pallidum* and Development of Their Stable Emulsion and Cream

**DOI:** 10.3390/biology14060625

**Published:** 2025-05-28

**Authors:** Shuoqi Wang, Li Wang, Yiqing Sun, Jia Yang, Wei Liu, Tingting Wu, Rui Jia

**Affiliations:** 1 College of Oceanography and Ecological Science, Shanghai Ocean University, Shanghai 201306, China; wangshuoqi0224@163.com (S.W.); orange709wl@163.com (L.W.); syq13818481629@163.com (Y.S.); yangjia0808@163.com (J.Y.); m220501178@st.shou.edu.cn (T.W.); 2 School of Environmental and Chemical Engineering, Shanghai University, Shanghai 200444, China; hsliuwei@shu.edu.cn; 3 Marine Biomedical Science and Technology Innovation Platform of Lingang Special Area, Shanghai 201306, China

**Keywords:** *Sargassum pallidum*, polyphenols, RAW264.7 cells, anti-inflammatory, functional cosmetics

## Abstract

Chronic inflammation poses a significant threat to physical health. The increasing prevalence of skin disorders is attributed to external factors such as environmental pollution and UV-induced stress. To address damaged skin, the cosmetic market increasingly favors the development of effective products containing natural active compounds. This study constitutes the initial examination of the anti-inflammatory properties of polyphenols extracted from *Sargassum pallidum*. The experimental results demonstrated that SPP significantly reduced intracellular levels of nitric oxide (NO), reactive oxygen species (ROS), and multiple inflammatory mediators. Furthermore, SPP exhibited an inhibitory effect on the mRNA expression of inflammation-related genes: compared to the control group, the mRNA levels of IL-1β, IL-6, TNF-α, iNOS, and COX-2 decreased by 4.94 ± 0.72-fold, 113.64 ± 27.46-fold, 1.40 ± 0.21-fold, 11.68 ± 3.90-fold, and 0.47 ± 0.11-fold, respectively (data presented as mean ± SD, * *p* < 0.05). SPP was incorporated into cosmetic formulations as a natural active ingredient to create emulsions and creams. Upon assessment of various skin parameters, these formulations demonstrated significant skincare benefits. In conclusion, SPP exhibits promising anti-inflammatory properties, making it a potential natural active ingredient for applications in cosmetics.

## Simple Summary

Skin disorders associated with persistent inflammation, often triggered by environmental pollution and ultraviolet (UV) exposure, are becoming increasingly prevalent. In response, natural active compounds are being widely explored for the development of effective skincare products. In this study, the anti-inflammatory activity of polyphenols extracted from *Sargassum pallidum* (SPP) was evaluated for the first time. SPP significantly attenuated oxidative stress and reduced the expression of inflammatory markers in cells. Furthermore, SPP was successfully incorporated into emulsion and cream formulations, which demonstrated improvements across multiple skin parameters. These findings indicate that SPP is a promising natural active ingredient with potential applications in the cosmetic industry.

## 1. Introduction

When an aberrant immune response occurs, it can lead to an inappropriate or excessive inflammatory reaction, triggering cell proliferation within the mononuclear phagocyte system [1]. Macrophages, as key regulators of the immune system, are instrumental in coordinating the inflammatory response via various pathways [1,2]. This activation is linked to the release of inflammatory mediators, which then trigger key signaling pathways involved in the inflammatory response [3,4]. Chronic inflammation poses a significant health risk; when left unchecked, it may lead to the onset of more serious medical conditions [5,6]. Furthermore, external factors such as environmental pollution and ultraviolet (UV) stress have contributed to the rising prevalence of skin conditions, including allergies, inflammation, and premature aging. In response to these escalating issues, the use of cosmetics containing natural active ingredients has gained popularity, as consumers seek greener and more secure alternatives [7,8].

Algae have abundant resources, and their metabolic compounds exhibit distinctive biological activities. Exploring the effective utilization of algal resources can unlock their potential applications.

Algae polyphenolic compounds are secondary metabolites derived from seaweeds, demonstrating a diverse array of biological activities, including skin-brightening, anti-aging, antidepressant, and antitumor effects. These attributes make them a compelling subject for scientific inquiry [9,10,11,12,13]. For example, a study by Kim et al. demonstrated that 6,6′-dieckol from *Ecklonia stolonifera* effectively inhibits the intracellular mRNA expression of pro-inflammatory cytokines [14]. Additionally, Han et al. demonstrated that phenolic compounds derived from *Sargassum horneri* possess anti-inflammatory properties, which are attributed to a reduction in intracellular NO and ROS levels, along with the suppression of the NF-κB/MAPK signaling pathway [15]. A substantial body of evidence supports the remarkable anti-inflammatory effects of brown algae polyphenolic compounds [16,17]. Given that current anti-inflammatory chemical drugs may have certain drawbacks and safety concerns, there is an urgent need to pursue and develop natural ingredient drugs that are safer and have fewer adverse effects.

*Sargassum pallidum*, which thrives along the Yellow Sea and Bohai Sea in China, possesses a variety of pharmacological effects, including the ability to lower blood pressure and regulate immune function. Additionally, it is recognized as a traditional Chinese medicinal plant [18]. Despite its potential, the exploration of *S. pallidum* metabolites has been largely undervalued. Further investigation is warranted to ascertain whether this substance can be developed into functional foods or drugs, thereby enhancing its utility and economic value. Su et al. [19] revealed that the antidepressant effects of active components from *Sargassum pallidum* are mediated through the inhibition of the ERK1/2/p38 inflammatory signaling pathway, which contributes to the reduction of intestinal inflammation levels. However, to date, the anti-inflammatory effects of polyphenols extracted from *Sargassum pallidum* remain unexplored.

Therefore, in this study, we established a cellular inflammation model using LPS-stimulated RAW264.7 cells to assess the anti-inflammatory effects of polyphenol extracts from *Sargassum pallidum* for the first time. These polyphenol extracts can be incorporated into skincare formulations and developed into products aimed at maintaining healthy skin and providing remedies for damaged skin.

## 2. Materials and Methods

### 2.1. Experimental Equipment and Reagents

The *Sargassum pallidum* was procured from the Chinese medicine market (Yantai, Shandong, China), then washed, placed in a ventilated hood and air-dried naturally, and ground into a fine powder. The powder was stored in a cool, dry environment to maintain its stability. Mouse mononuclear macrophage leukemia cells (RAW264.7 cells) were obtained from Shanghai Chaorui Biotechnology Co., LTD. (Shanghai, China). Lipopolysaccharides (LPSs) were obtained from Sigma Aldrich (Shanghai, China). Dulbecco’s modified eagle medium (DMEM), dimethyl sulfoxide (DMSO), penicillin-streptomycin (P/S), and phosphate-buffered saline (PBS) were obtained from Nanjing BioChannel Biotechnology Co., LTD. (Nanjing, China). Fetal bovine serum (FBS), cell counting kit-8 (CCK-8), NO assay kit, and 2′,7′-Dichlorodihydrofluorescein diacetate (DCFH-DA) were obtained from Beyotime Biotechnology Co., LTD. (Shanghai, China). The enzyme-linked immunosorbent assay kit (ELISA) for interleukin-1β (IL-1β), interleukin-6 (IL-6), tumor necrosis factor-α (TNF-α), and prostaglandin E2 (PGE2) were obtained from Beijing winter song Boye Biotechnology Co., LTD. (Beijing, China). RNAprep Pure Cell Kit was obtained from TIANGEN Biotech Co., LTD. (Beijing, China). cDNA synthesis kit and TB Green PCR Premix kit were obtained from Takara Biomedical Technology (Beijing) Co., LTD. (Beijing, China). All other chemicals and reagents were of analytical grade.

### 2.2. Determination of Phenolic Compounds

The extraction experiments were conducted by the methodology outlined by Safdar et al., with the incorporation of minor modifications [20]. In brief, algal powder from *S. pallidum* was weighed at a ratio of 1:20 (M/V), and a 30% (V/V) ethanol solution was used as the extraction solvent, utilizing ultrasonic-assisted extraction. The purification of the extracts was performed using ADS-21 Adsorption Resin, with the eluent collected in segments for subsequent thin-layer chromatography analysis. Additionally, the Folin–Ciocalteu method was employed to quantify the polyphenol content.

### 2.3. Culture RAW264.7 Cell

The RAW264.7 cell line was cultured in a humidified incubator at 37 °C with 5% CO_2_ using high-glucose DMEM supplemented with 10% FBS and 1% P/S. This cell line exhibits rapid growth and requires passaging when the cellular density reaches 80% to 90%.

### 2.4. Cytotoxicity Assay

The optimal concentration of SPP was evaluated by performing a CCK-8 assay to assess cell viability [21]. The RAW264.7 cells, in the logarithmic growth phase, were inoculated into 96-well culture plates at a density of 1 × 10^4^ cells/well and cultured for 24 h. Following this, different concentrations of SPP (0, 10, 25, 50, 75, 100, 150, and 200 μg/mL) were introduced to continue the culture. Following the manufacturer’s instructions, CCK-8 was added to the 96-well plates. The optical density (OD) of individual wells in the plate at 450 nm was then measured using an ELISA reader (Bio-Rad, Hercules, California, USA).

### 2.5. Intracellular NO Levels

NO levels were quantified using the Griess method in accordance with the manufacturer’s guidelines [22]. A control group was set up to evaluate the impact of different concentrations of lipopolysaccharides (LPS) on NO expression in RAW264.7 cells. In addition, RAW264.7 cells were pre-treated with SPP for 8 h at concentrations of 25.0, 50.0, and 75.0 μg/mL, after which 2 μg/mL of LPS was added, and incubation was extended for another 16 h. Subsequently, the NO levels were measured to assess the protective impact of SPP on LPS-stimulated RAW264.7 cells.

### 2.6. Detection of Inflammation-Related Mediators

The expression levels of IL-1β, IL-6, TNF-α, and PGE2 were quantified in RAW264.7 cells [23]. As detailed in Section 2.4, the cells were inoculated, and 100 μL of horseradish peroxidase (HRP)-labeled detection antibody was added to each well, excluding the blank wells. In accordance with the manufacturer’s instructions, the ELISA was employed to determine the levels of IL-1β, IL-6, TNF-α, and PGE2 following the administration of SPP.

### 2.7. Measurement of Reactive Oxygen Species (ROS) Levels

The expression levels of ROS in RAW264.7 cells were determined using the DCFH-DA probe [24]. The cells were inoculated as described in Section 2.5. DCFH-DA was added to the cell plate at a final concentration of 10 μmol/L and transferred to the cell culture incubator for 40 min of dark incubation. The level of cellular ROS was then assessed based on the fluorescence intensity detected by a fluorescent enzyme labeler with an excitation wavelength of 488 nm and an emission wavelength of 525 nm (BIOTEK, Winooski, VT, USA).

### 2.8. RT-PCR Analysis

The influence of SPP on the mRNA expression of IL-1β, IL-6, TNF-α, inducible nitric oxide synthase (iNOS), and cyclooxygenase-2 (COX-2) in RAW264.7 cells was assessed. The relevant gene sequences were retrieved from the NCBI database, and primer sequences were subsequently designed using Primer-BLAST. The specific primer sequences are shown in Table 1. In accordance with the manufacturer’s instructions, total cellular RNA was extracted using TRIZOL reagent (TIANGEN, Beijing, China). Complementary DNA (cDNA) for RT-qPCR was synthesized using a reverse transcription kit (TaKaRa, Kasatsu, Japan), with GAPDH serving as the reference standard.

### 2.9. Preparation of Emulsions and Creams

Following the method proposed by Liu et al. [25], the formulations for the emulsions and creams were developed. Based on the formulations provided in the Supplementary Material Tables S1 and S2, the ingredients of the oil phase were combined in beaker A and heated to 80 °C to ensure complete dissolution. Concurrently, the ingredients of the aqueous phase were added to beaker B and heated to 90 °C for 20 min. The solution in beaker A was then mixed with that in beaker B and homogenized for 5 min before being cooled to approximately 48 °C. Subsequently, the ingredients of the additive phase were incorporated, and the mixture was further homogenized. The emulsions and creams were produced using SPP as a natural plant ingredient. Additionally, the emulsions and creams were evaluated for physicochemical indicators, including centrifugal stability, thermal stability, cold stability, irritation, and preservative properties.

### 2.10. Evaluating the Effectiveness of Emulsions and Creams

Volunteers with varying skin types were selected for the study, with a 3 × 3 cm area on their cheeks designated as the test area. Prior to the test, subjects were instructed to cleanse their faces and apply an appropriate quantity of the product to ensure complete absorption. A multifunctional smart skin tester was utilized to measure the water content, oil content, skin erythema index, and elasticity index of the subjects’ faces. Data were recorded at ten-day intervals.

### 2.11. Statistical Analysis

The data are presented as the mean ± standard error (S.E.), and an ANOVA test was employed to statistically compare the mean values using SPSS 26.0 statistical package and GraphPad Prism 8. All experiments were performed in triplicate, and the differences were considered statistically significant at *p* < 0.05.

## 3. Results

### 3.1. TLC Polyphenol Characterization

**Figure 1:**
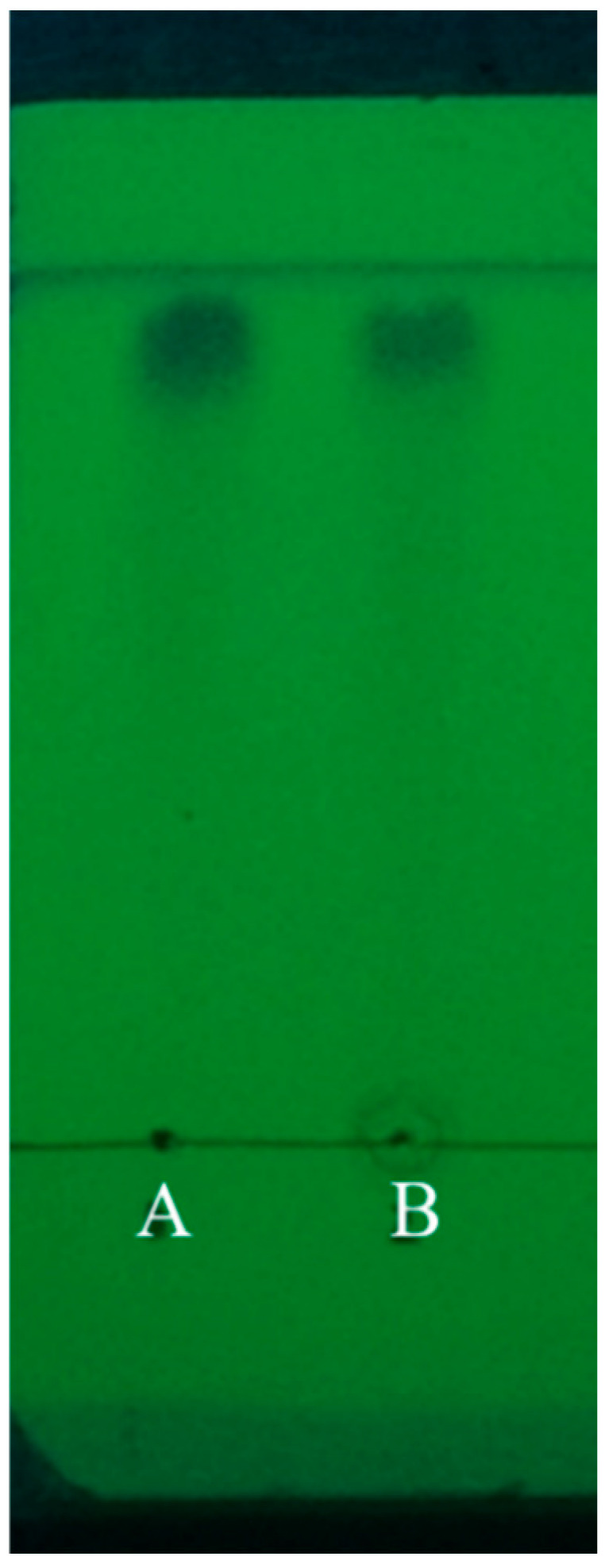
TLC of polyphenols from *Sargassum pallidum*. (A) Represents the gallic acid standard, (B) represents the purified *S. pallidum* polyphenols.

The polyphenol extraction rate achieved through ultrasound-assisted extraction was 6.384 mg/g, and the purity of polyphenols purified using ADS-21 adsorbent resin was 41.73%. Thin-layer chromatography (TLC) was employed for the identification and characterization of the purified *S. pallidum* polyphenols, as shown in Figure 1. Using gallic acid as the standard, it was observed that the distance traveled by the sample spots from the origin to the front edge of the solvent was nearly identical to that of the gallic acid spots. Additionally, upon the addition of the FeCl_3_ solution, the polyphenols exhibited a characteristic color reaction, confirming that the purified *S. pallidum* polyphenols possess the typical properties of phenolics.

### 3.2. Effect of SPP on the Viability of RAW264.7 Cells

**Figure 2:**
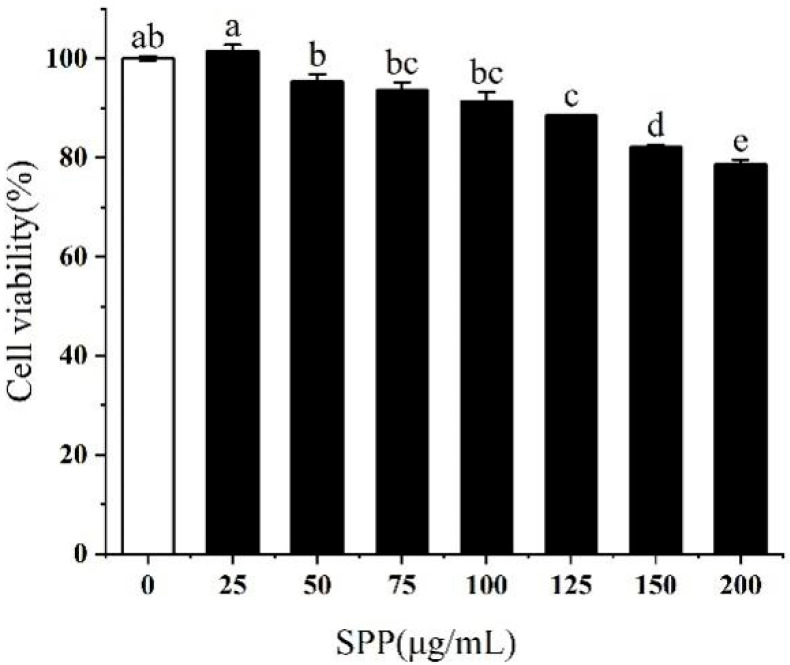
Effect of SPP on the viability of RAW264.7 cells. The experiments were repeated in triplicate. The different letters in the bars represent significant differences (*p* < 0.05).

The impact of SPP on the activity of RAW264.7 cells was investigated. The results revealed that SPP concentrations below 75 μg/mL were essentially non-toxic to RAW264.7 macrophages. However, a reduction in cell viability to 91.3% was noted at a concentration of 100 μg/mL (Figure 2). The optimal concentrations of SPP were determined to be 25, 50, and 75 μg/mL, to ensure that the cells retained normal physiological activity.

### 3.3. The Anti-Inflammatory Effects of SPP

**Figure 3:**
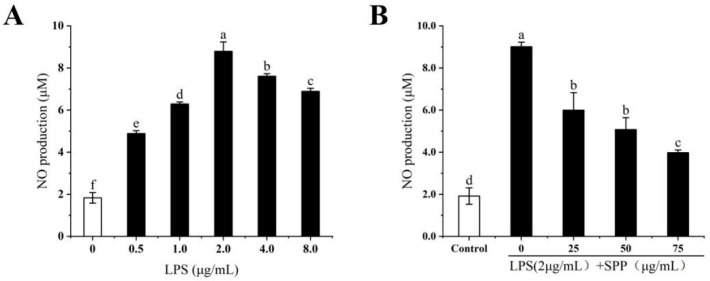
Effect of different concentrations of LPS (**A**) and SPP (**B**) on the NO content of RAW264.7 cells. The experiments were repeated in triplicate. The different letters in the bars represent significant differences (*p* < 0.05).

LPS stimulation of macrophages induces the release of substantial amounts of NO, which is often considered a marker of macrophage activation [17]. The anti-inflammatory effect of SPP was assessed by measuring NO levels in RAW264.7 cells (Figure 3). Measurements of NO levels in RAW264.7 cells stimulated with different concentrations of LPS revealed that 2 μg/mL LPS represents the optimal induction concentration. The effect of SPP on the inhibition of LPS-induced NO synthesis in RAW264.7 cells was investigated. The production of intracellular NO was markedly diminished by SPP pre-treatment at concentrations of 25, 50, and 75 μg/mL compared to the induction group, exhibiting a concentration-dependent pattern. The observed difference was statistically significant (*p* < 0.05).

### 3.4. Effect of SPP on the Expression of Relevant Inflammatory Elements (TNF-α, IL-1β, IL-6, PGE2)

**Figure 4:**
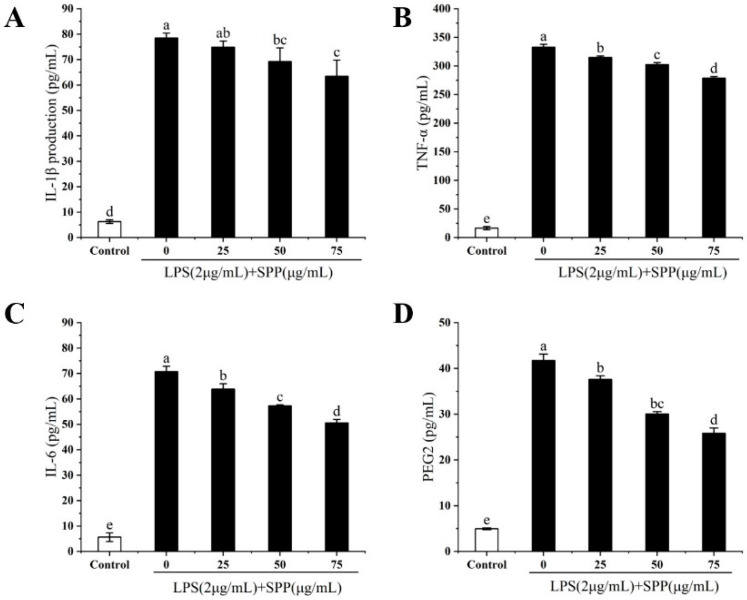
Effect of different concentrations of SPP on inflammatory elements in RAW264.7 cells. The figure depicts the effects of SPP on the production of IL-1β (**A**), IL-6 (**B**), TNF-α (**C**), and PGE2 (**D**) in RAW264.7 cells. The different letters in the bars represent significant differences (*p* < 0.05).

The overexpression of elements associated with inflammation activates intracellular signals that regulate inflammatory processes, leading to excessive inflammatory manifestations [16]. The anti-inflammatory effects of SPP were assessed by quantifying the expression levels of TNF-α, IL-1β, IL-6, and PGE2 in RAW264.7 cells (Figure 4). In comparison to the LPS-induced group, the expression levels of intracellular TNF-α, IL-1β, IL-6, and PGE2 were markedly diminished following pre-treatment with SPP, exhibiting a concentration-dependent effect. These results indicate that SPP may effectively inhibit the overproduction of pro-inflammatory factors.

### 3.5. Effect of SPP on Intracellular ROS Generation

**Figure 5:**
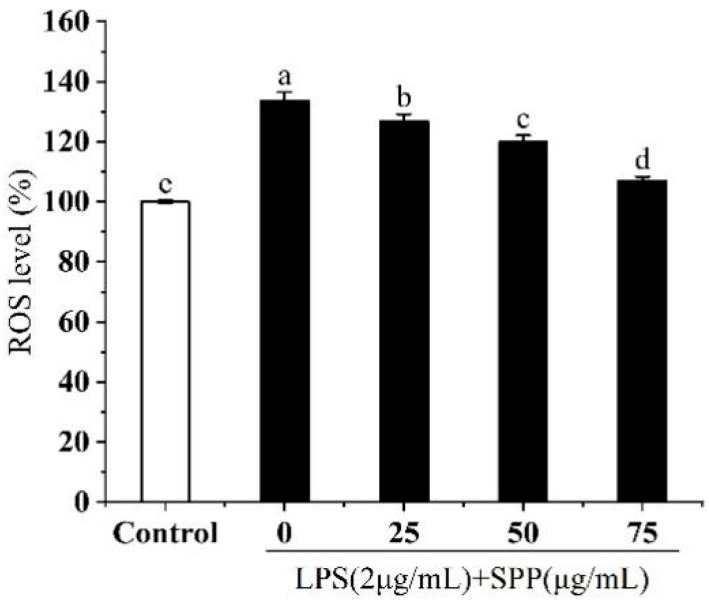
Effect of different concentrations of SPP on ROS levels in RAW264.7 cells. The experiments were repeated in triplicate. The different letters in the bars represent significant differences (*p* < 0.05).

Excessive accumulation or depletion of ROS can destabilize redox homeostasis, potentially affecting numerous cellular signaling pathways and ultimately leading to cellular dysfunction and the development of various pathological conditions [26]. The DCFH-DA probe is oxidized upon binding to ROS, resulting in the generation of a green fluorescent substance that is proportional to the level of ROS (Figure 5). Compared to the control group, following stimulation with LPS, a significant increase in ROS levels was observed. Pre-treatment with SPP has been shown to markedly diminish the generation of excess ROS in cells.

### 3.6. The IL-1β, IL-6, TNF-α, iNOS, and COX-2 mRNA Expressions

**Figure 6:**
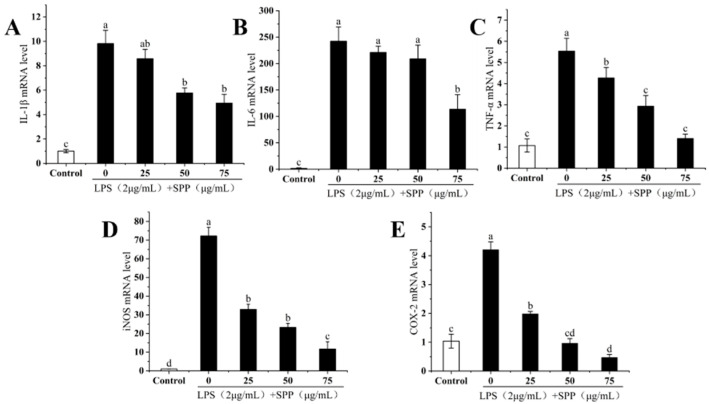
Effect of SPP on the expression of IL-1β (**A**), IL-6 (**B**), TNF-α (**C**), iNOS (**D**), and COX-2 (**E**) mRNA. The experiments were repeated in triplicate. The different letters in the bars represent significant differences (*p* < 0.05).

Inflammatory responses rely on various signaling pathways, cellular expression factors, and antigen receptors. IL-1β, IL-6, and TNF-α are classified as pro-inflammatory factors [15]. The effects of SPP on the mRNA expression levels of IL-1β, IL-6, TNF-α, iNOS, and COX-2 in RAW264.7 cells were further investigated. SPP was found to markedly inhibit the elevated expression of TNF-α, IL-1β, and IL-6 in RAW264.7 cells (Figure 6A–C), with the suppressive effect becoming more evident at higher concentrations. iNOS and COX-2 are marker enzymes that reflect the degree of inflammatory expression and serve as inflammatory regulators. Elevated expression levels of iNOS and COX-2 promote the secretion of NO and PGE2, respectively [15]. SPP demonstrated the ability to reduce the mRNA expression levels of iNOS and COX-2 in a concentration-dependent manner (Figure 6D,E). Consequently, the anti-inflammatory effect of SPP is achieved through the inhibition of the mRNA expression of inflammatory cytokines and key enzymes.

### 3.7. Testing the Physicochemical Properties of Emulsions and Creams

**Figure 7:**
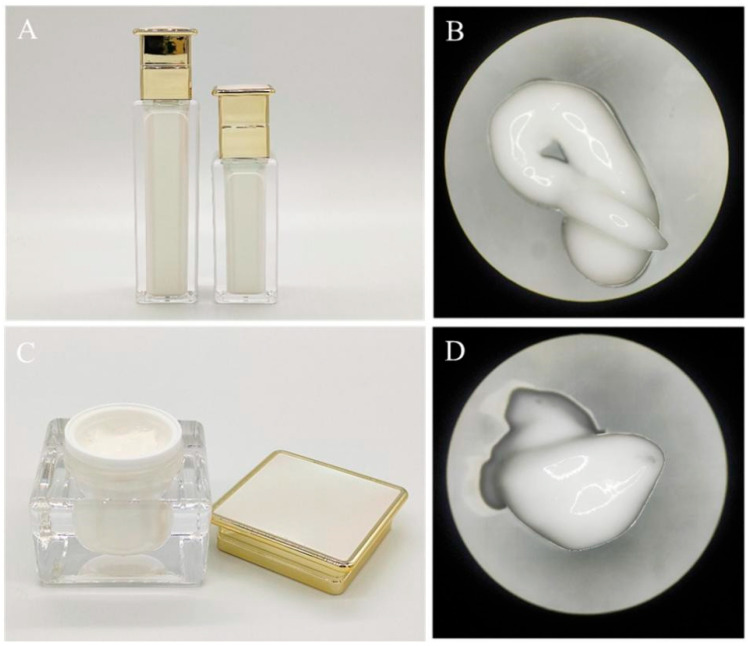
(**A**,**B**) The emulsions product and microstructure; (**C**,**D**) the creams product and microstructure. The experiments were repeated in triplicate.

The physicochemical properties of the emulsions and creams were evaluated to confirm their safety and stability. Microscopic examination revealed that the emulsions and creams exhibited a fine, glossy texture with uniform distribution and no visible particles (Figure 7). These findings indicate that the prepared emulsions and creams comply with established cosmetic safety standards.

### 3.8. Skin Benefits of Emulsions and Creams

**Figure 8:**
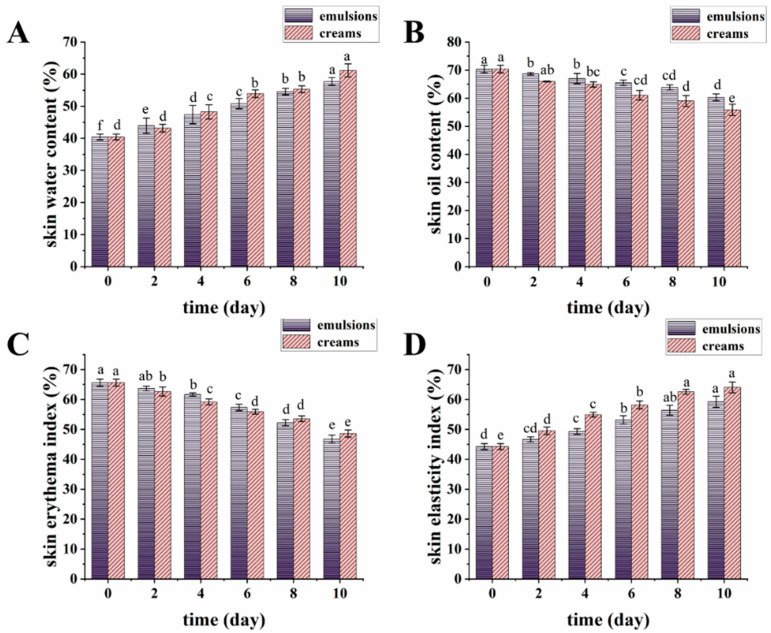
The results on measuring skin parameters of emulsions and creams. (**A**) Skin water content measurements. (**B**) Skin oil content measurements. (**C**) Skin erythema index measurement. (**D**) Skin elasticity index measurements. The experiments were repeated in triplicate. The different letters in the bars represent significant differences (*p* < 0.05).

**Figure 9:**
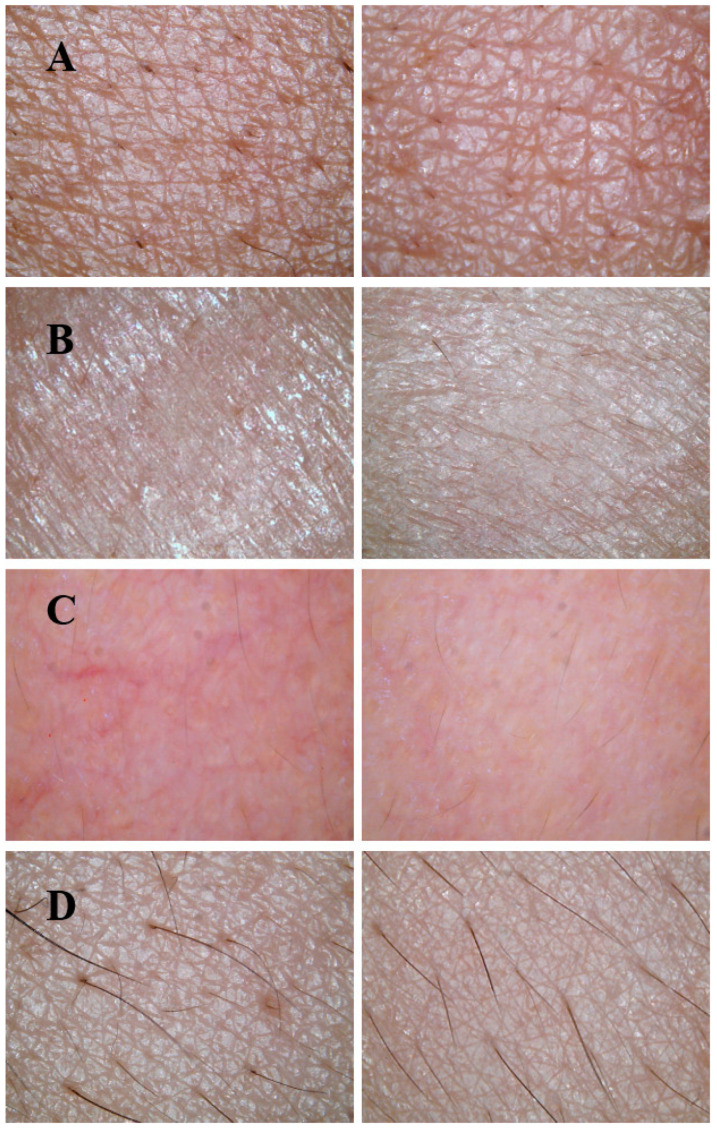
Skin condition of subjects before and after using lotions and creams. (**A**) Volunteer with dry skin. (**B**) Volunteer with oily skin. (**C**) Volunteer with sensitive skin. (**D**) Volunteer with normal skin. Note: The picture on the left is before use and the picture on the right is after use.

The skin index of the volunteers was measured using a multi-functional smart skin tester (Figure 8), and photographs were taken to document the skin condition of volunteers on day 0 (before use) and day 10 (after use) (Figure 9).

From the skin index levels over the study period, it can be observed that the initial water content was (40.74 ± 1.92)%, indicating a relatively low level of hydration. This value gradually increased over time, reaching (57.71 ± 1.18)% for emulsions and (61.17 ± 2.04)% for creams by the 10th day. The subjects had an initial oil index of (70.38 ± 1.35)%, which decreased to (55.87 ± 2.01)% for creams and (60.30 ± 1.22)% for emulsions on the 10th day. The initial skin erythema index was (65.7 ± 1.17)%, with the erythema index of the emulsion on the eighth day being lower than that of the cream, indicating that the emulsion demonstrated a superior capacity to improve skin erythema. The initial skin elasticity was (44.25 ± 1.01)%, showing a gradual improvement with continued use. By the 10th day, skin elasticity was observed to be (59.26 ± 1.86)% for emulsions and (64.06 ± 1.83)% for creams. In conclusion, the emulsions and creams exhibited positive skincare efficacy.

As shown in Figure 9, the skin of volunteers with dry skin appeared dry and dehydrated before product application. However, after 10 days of use, the skin became hydrated, full, and glossy with improved tension, indicating that the lotion and cream exhibited excellent moisturizing properties. For volunteers with oily skin, the initial skin condition showed excessive oil production, but after using the lotion and cream, the oiliness was significantly reduced, demonstrating the products’ effective oil-control capabilities. Volunteers with sensitive skin initially displayed visible redness, but after 10 days of application, the redness was greatly diminished, and the skin condition returned to near-normal, proving the lotion and cream’s ability to soothe and stabilize sensitive skin. Volunteer 4 (normal skin) had poor skin elasticity and rough texture before the trial. After 10 days of use, the skin became smoother and more delicate, indicating that the lotion and cream significantly improved skin elasticity. Overall, the results demonstrate that the lotion and cream effectively address various skin conditions, including hydration, oil control, sensitivity, and elasticity enhancement.

## 4. Discussion

Lipopolysaccharides can activate macrophages through the cellular signal transduction system, prompting the body to synthesize and secrete a variety of pro-inflammatory mediators. These mediators, in turn, elicit a cascade of inflammatory responses [27,28]. The skin, serving as the first line of defense for the immune system, is continuously exposed to external irritants, including oxidative stress-inducing factors such as toxins and pathogens. Additionally, the skin’s inflammatory response represents the body’s innate defense mechanism against these irritants, but chronic exposure to oxidative stress can lead to premature aging, compromised barrier integrity and reduced immunological resilience [27,29,30,31]. Recent studies highlight that polyphenols exert potent anti-inflammatory effects, not only by modulating gene expression but also by directly interacting with receptors involved in the activation of inflammatory pathways, such as MAPK, NF-κB, and Nrf2 [32,33,34].

In this study, the RAW264.7 cytotoxicity assay was conducted to ascertain the optimal SPP dose concentrations, identified as 25, 50, and 75 μg/mL [35,36]. Nitric oxide (NO) is a key mediator of inflammation, playing a pivotal role in various physiological and pathological processes, including neurotransmission, immune response, and apoptosis [27,37,38]. LPS stimulation of RAW264.7 cells induced the release of considerable quantities of NO, indicative of an exacerbated inflammatory response. SPP was observed to reduce intracellular NO release in a dose-dependent manner. ROS are involved in inflammatory diseases and immune regulation, however, excessive ROS production or prolonged action can pose a significant threat to cells [39,40]. Following stimulation with LPS, the ROS level increased to 133.71%. In contrast, pre-treatment with SPP markedly suppressed the generation of intracellular ROS. The application of 75 μg/mL SPP reduced cellular ROS levels to 107.1%, thereby attenuating cellular damage caused by ROS proliferation. Elevated levels of pro-inflammatory cytokines within the organism indicate an augmented inflammatory response, and additionally, the perpetuation of inflammation is a contributing element in the progression of conditions, including tumorigenesis and cardiovascular complications [41,42]. The expression amounts of inflammatory mediators were examined. SPP was observed to significantly diminish the intracellular concentrations of TNF-α, IL-1β, IL-6, and PGE2. This suggests that SPP effectively inhibits the secretion of pro-inflammatory mediators, demonstrating its efficacy. Additionally, the mRNA expression levels of IL-1β, IL-6, TNF-α, iNOS, and COX-2 in the cells were quantified. The results showed that SPP reduced the mRNA expression of these inflammatory elements. iNOS and COX-2 are critical enzymes that promote the production of NO and PGE2. A reduction in the mRNA expression of iNOS and COX-2 results in a corresponding decline in the production of NO and PGE2, which attenuates the inflammatory response. Consequently, the anti-inflammatory effects of SPP are reflected in the modulation of mRNA expression levels associated with inflammatory mediators and active enzymes.

The capacity of the skin to retain a youthful and elastic quality is largely attributed to its high water content. The natural aging process, along with certain external factors such as cigarette smoke, kitchen smoke, and UV rays, can reduce the skin’s water retention capacity, thereby accelerating the aging process. It is evident that water retention is of paramount importance for maintaining skin health [43,44]. Additionally, aging results in a decrease in elastin and collagen within the skin, which contributes to the formation of wrinkles. Recent studies have emphasized that plant-derived bioactives—rich in polyphenols, polysaccharides, and peptides—can reinforce the skin’s antioxidant defenses, regulate inflammatory pathways, and support barrier integrity, thus helping to sustain hydration and delay aging-related deterioration [30]. Moreover, skin is not merely a passive physical barrier but functions as an active immune organ. The complex crosstalk between the nervous and immune systems has been recognized as a crucial modulator in inflammatory skin conditions, which can influence both skin aging and overall health maintenance [31]. The penetration of cosmetic active ingredients through the epidermis facilitates their absorption into the body, thereby slowing the rate of skin aging and helping to maintain healthy, youthful skin [45,46,47]. In the present study, the anti-inflammatory properties of SPP were explored by incorporating the compound into cosmetic formulations prepared as emulsions and creams. The emulsions and creams underwent rigorous testing for a range of physicochemical properties and were found to comply with all relevant cosmetic safety standards [48,49,50]. Furthermore, the water content, oil content, redness index, and skin elasticity of the face were evaluated, demonstrating that emulsions and creams formulated with SPP as the natural active ingredient exhibited excellent efficacy in improving dermal conditions. In summary, SPP shows prominent anti-inflammatory activity and can be considered a natural anti-inflammatory active substance for application in cosmetics and food.

## 5. Conclusions

In this investigation, polyphenolic substances from *Sargassum pallidum* (SPP) were extracted, and their potential anti-inflammatory properties were evaluated for the first time. The results demonstrate that SPP not only reduces intracellular NO secretion in RAW264.7 cells but also significantly inhibits excessive ROS formation. Furthermore, SPP was observed to inhibit the release of TNF-α, IL-1β, IL-6, and PGE2 in the cells while simultaneously downregulating the mRNA expression levels of IL-1β, IL-6, TNF-α, iNOS, and COX-2. Additionally, the incorporation of SPP as a natural active ingredient into cosmetic formulations has resulted in the generation of emulsions and creams that exhibit remarkable capabilities in moisturizing, oil control, skin sensitivity relief, and skin elasticity enhancement. In conclusion, the anti-inflammatory potential of SPP is significant and should not be overlooked. It may be developed as an anti-inflammatory drug or effective cosmetic, capitalizing on the resourcefulness and economic benefits of *S. pallidum*.

## Figures and Tables

**Table 1:** Primer sequences.

Gene	Primer Sequences
IL-1β	F: 5′-TTCAGGCAGGCAGTATCACTC -3′
	R: 5′-GAAGGTCCACGGGAAAGACAC-3′
IL-6	F: 5′-TCCATCCAGTTGCCTTCTTG-3′
	R: 5′-AAGCCTCCGACTTGTGAAGTG-3′
TNF-α	F: 5′-ACTGGCAGAAGAGGCACTCC-3′
	R: 5′-GCCACAAGCAGGAATGAGAA-3′
iNOS	F: 5′-CCTCCTCGTTCAGCTCACCT-3′
	R: 5′-CAATCCACAACTCGCTCCAA-3′
COX-2	F: 5′-CCTGGTGAACTACGACTGCTA-3′
	R: 5′-AGTGGAGAACGTCTTCAGATGAG-3′
GAPDH	F: 5′-GTGAAGGTGACAGCAGTCGGTT-3′
	R: 5′-GAAGTGGGGTGGCTTTTAGGA-3′

## Data Availability

Data are available from the corresponding author upon reasonable request.

## Supplementary Materials

The following supporting information can be downloaded at: https://www.mdpi.com/article/10.3390/biology14060625/s1, Table S1. Moisturising and soothing lotion formulations. Table S2. Whitening and soothing cream formulations.
