# Supplementary material for: Evaluation of the Bioactivity of Phenolic Compounds from the Sargassum pallidum and Development of Their Stable Emulsion and Cream
Source: Biology (Basel). 2025 May 28;14(6):625. doi: 10.3390/biology14060625 (PMC12189737; doi:10.3390/biology14060625)
Supplement: Supplementary file 1 [file biology-14-00625-s001.zip › biology-3669186-supplementary.pdf]

Table S1. Moisturising and soothing lotion formulations

Component	Ingredient Name	Mass Fraction %	Function
A) Oil-phase	Cetearyl alcohol	1.0	Thickener /Emollient
	Silicone oil	3.0	Emulsifier
	Vitamin E	0.3	Emollient
	Caprylic/capric triglyceride	5.0	Emollient
	Cetearyl olivate、Sorbitan olivate	2.0	Emulsifier
	No. 26 white oil	1.0	Moisturizer
	Span 60	1.0	Emulsifier
B) Aqueous phase	(EG) Acrylamide/Sodium Acryloyldimethyltaurate Copolymer /Polysorbate 80	0.5	Emulsifier /Thickener
	1,3-Butanediol	3.0	Moisturizer
	Sodium hyaluronate	0.02	Thickener
	Glycerol	4.0	Moisturizer
	Xanthan gum	0.05	Thickener
	Betaine	1.0	Moisturizer
	Centella Asiatica Extract	3.0	Smooth/Emollient
	Semen Coicis Extrac	5.0	Whitening/Emollient
	Triethanolamine	0.1	Neutralizer
	EDTA-2Na	0.1	Chelating Agent
	Deionized water	To 100	To 100
C) Adding phase	Alpha-Bisabolol	0.2	Sensitizers/Anti-inflammatory agents
	Nicotinamide	0.8	Whitening and Wrinkle
	Algae Extract	0.3	Active Ingredient
	3-o-ETHYL ASCORBICACID	0.5	Antioxidant
	Ashland	0.7	Preservative
	Parfum	0.1	Aromatics

Table S2. Whitening and soothing cream formulations

Component	Ingredient Name	Mass Fraction %	Function
A) Oil-phase	Shea Butter	0.8	Emollient
	Glycerol Monostearate	0.5	Emulsifier
	Vitamin E	0.2	Emollient
	Caprylic/capric triglyceride	5.0	Emollient
	2-Ethylhexyl palmitate	2.0	Emollient
	Silicone oil	1.0	Emulsifier
	Cetearyl Alcohol	1.5	thickening /Emollient
	Dicaprylyl Carbonate	2.0	Emollient
	Glyceryl stearate and PEG-100 Stearate	2.0	Emulsifier
	Plant squalane	4.0	Emollient
B) Aqueous phase	Glycerol	3.0	Moisturizer
	Propylene Glycol	4.0	Moisturizer
	Sodium hyaluronate	0.03	Moisturizer
	Sodium polyacrylate (G57)	0.5	Thickener
	Fucose	1.0	Moisturizer
	Carbomer 941	0.3	Thickener
	Triethanolamine	0.1	Neutralizer
	Centella Asiatica Extract	3.0	Smooth/Emollient
	Witch hazel extract	5.0	Firming /Emollient
	EDTA-2Na	0.05	Chelating Agent
	Deionized water	To 100	To 100
C) Adding phase	Alpha-Bisabolol	0.2	Sensitizers/Anti-inflammatory agents
	Nicotinamide	0.8	Whitening and Wrinkle
	Laminaria	0.3	Active Ingredient
	α- Arbutin	0.5	Antioxidant
	Ashland	0.7	Preservative
	Essence	0.1	Aromatics
